# Doctor-Patient Relationship in the Eyes of Medical Professionals in China During the COVID-19 Pandemic: A Cross-Sectional Study

**DOI:** 10.3389/fpsyt.2021.768089

**Published:** 2021-10-28

**Authors:** Yanan Zhou, Winson Fu Zun Yang, Yuejiao Ma, Qiuxia Wu, Dong Yang, Tieqiao Liu, Xiaoming Wu

**Affiliations:** ^1^Department of Psychiatry, Hunan Brain Hospital (Second People's Hospital of Hunan Province), Changsha, China; ^2^Department of Psychiatry, National Clinical Research Center for Mental Disorders, The Second Xiangya Hospital of Central South University, Changsha, China; ^3^Department of Psychological Sciences, College of Arts and Sciences, Texas Tech University, Lubbock, TX, United States; ^4^Department of Cardiovascular Surgery, The Second Xiangya Hospital, Central South University, Changsha, China

**Keywords:** COVID-19, doctor-patient relationship, public education, media reports, medical resources, communication

## Abstract

**Background:** Doctor-patient relationship (DPR) is very important for patient outcomes, especially during a public health emergency like the COVID-19 pandemic. However, few studies have evaluated DPR and related sentiments from medical professionals' perspectives. Thus, the aim of the study is to provide a better understanding of DPR from medical professionals' perspectives during the COVID-19 pandemic in China.

**Methods:** A total of 979 medical professionals, including doctors, nurses, technicians, and other workers have completed a series of questionnaires to evaluate their attitudes toward DPR, trust, violence against doctors, factors that affected and improved DPR, and the importance of these factors on DPR. Analyses of variances (ANOVA) and linear regressions were used to analyze the effects of the pandemic, demographic variables, and various elements on DPR.

**Results:** One-way ANOVA revealed a significant effect of education on recent DPR [*F*_(2, 976)_ = 6.17, *p* < 0.001 and trust at *F*_(2, 976)_ = 9.54, *p* < 0.001], indicating that individuals with higher level of education (bachelor's degree, Master's degree and above) showed poorer recent DPR and lower level of trust. The level of hospital also showed a significant effect on trust [*F*_(5, 973)_ = 3.79, *p* = 0.0021]. Cochran's Q test revealed a significant difference in factors that affected [Q_(11)_ = 3,997.83, *p* < 0.001] and improved [Q_(8)_ = 3,304.53, *p* < 0.001] DPR. Backward stepwise linear regressions revealed predictors for changes during [*F*_(9, 969)_ = 21.17, *p* < 0.001, *R*^2^ = 0.16], shortly after [*F*_(7, 971)_ = 54.98, *p* < 0.001, *R*^2^ = 0.28], and long after [*F*_(10, 968)_ = 37.83, *p* < 0.001, *R*^2^ = 0.29] the pandemic.

**Conclusions:** Medical professionals' perceptions of DPR is important as they provide basis for the improvement in working environment of medical professionals and hospital visiting experience of patients, as well as healthcare policy making and preparation for future public health emergencies.

## Introduction

The year 2020 has experienced an international public health crisis, the pandemic of novel coronavirus disease 2019 (COVID-19). To date, the disease caused by SARS-Cov-2 has affected more than 70 countries worldwide (1). Due to its highly contagious nature (2), diverse clinical manifestations (3), and long incubation period (4), this pandemic poses a serious threat to human health. As a result, many other fields, such as public psychological health (5) and well-being (6), as well as medical systems (7–9), have been affected by this crisis. The doctor-patient relationship (DPR) is one of the affected aspect (10, 11).

DPR is important for good medical practice, as it influences compliance with treatment and shapes subjective perception about the doctor, patient, and medical services (12). During a health crisis like the COVID-19 pandemic, positive DPR is even more important as it directly influences the overall psychological and physical health of people. A recent study reported that people's confidence in medical services and satisfaction with healthcare information provided to the public directly affected the mental and psychological health of the public during the COVID-19 pandemic (13).

In China, the rapidly evolving pandemic has led to concerns in the entire health-care system and brought unprecedented challenges to DPR in China (14, 15). Many medical professionals were sent to the frontline in their counties or cities or were sent to Hubei province to meet the high and urgent demand for medical resources (16, 17). Overburdened hospitals and medical professionals were faced with a large influx of patients with COVID-19, affecting the routine care activities in the hospitals (18). Strict preventive strategies, such as physical distancing, face mask, and personal protective equipment to prevent virus transmission, created barriers to effective doctor-patient communication, eventually leading to a reduced trust in doctors and other healthcare workers (19). Moreover, given the lack of curative interventions, this time of uncertainty brought stress on both medical workers and patients (20), which might lead to the worsening of DPR.

Despite the negative impacts of COVID-19 on DPR, medical workers in China bravely rose to the challenge; many of them worked voluntarily at the frontline against the pandemic (21). Their professionalism affected society's perception of medical professionals and strengthened mutual trust and understanding between patients and medical workers (22–24), with many reports referring to medical professionals as heroes and praising their hard work through media, which in turn led to improvement of DPR. With the above factors affecting the DPR in China, the impact of the COVID-19 epidemic on China's DPR has led to heated debate (25).

As the COVID-19 pandemic is a health crisis, medical professionals shouldered the burden of great responsibility and heavy workload more painfully than most other groups (26). According to media reports, many Chinese medical professionals expressed that DPR has been significantly improved during the COVID-19 pandemic due to better patient compliance, as well as increased trust and respect from patients. A recent study reported improved DPR during the pandemic from the patients' perspective (27). However, quantitative empirical research on the perception of DPR among Chinese medical workers during the pandemic has not yet been carried out.

Therefore, in the present study, we used empirical investigation methods to examine the perception of DPR in Chinese medical workers. We also investigated factors predicting changes in DPR during the pandemic and approaches that could improve DPR. Based on previous literature, we hypothesized that medical workers might have a positive view of DPR during the pandemic; we also predicted that several factors such as communication, media, and understanding of medical work will be main predictors for DPR. Hopefully, this study will provide a forward-looking perspective for the influence of the crisis on DPR as well as key points for clinicians and even policy makers to help develop a more constructive and positive DPR.

## Methods

### Respondents

This is a cross-sectional, retrospective, anonymous study carried out between March 12 and March 30, 2020. In this online study, we used convenience and snowball sampling approaches to recruit respondents in China via advertisement posted on various websites and social media. The inclusion criteria were as follows: (1) aged 18 years and above, (2) engaged in medical works, (3) living in China, and (4) still working during the pandemic. Interested respondents were given a hyperlink to Questionnaire Star (https://www.wjx.cn), a professional website for surveys, with an ethics approved consent form in the first page. Respondents who provided consent via the electronic informed consent form were able to proceed to the demographic survey and the DPR questionnaire. This study was approved by the Ethics Committee of the Second Xiangya Hospital of Central South University (No. LYE2020041).

### Measures

#### Socio-Demographics

Socio-demographic information, including age, gender, level of education, monthly income, residency, position, title, years of working, level of hospitals they work at, and the department they work at during the pandemic, was collected and recorded for all respondents.

#### General Perception of DPR

Respondents' general perception of DPR before and during the COVID-19 pandemic was assessed through a series of questions like “What do you think of the doctor-patient relationship in China in recent years/during the COVID-19 pandemic?” The answers were provided with the use of a 5-point Likert scale ranging from 1 (extremely adversarial) to 5 (very harmonious). Respondents were also asked about their views of the short-term and long-term trends of DPR changing after the pandemic. The answer of these two questions were also rated on a 5-point Likert scale ranging from 1 (significantly worsened) to 5 (significantly improved). Change in DPR was computed by taking the centering scores around the 0-point, followed by taking the difference between the DPR during the pandemic and before the pandemic. In other words, the score of 0 indicated no change in the perception of DPR, while scores above 0 indicated improvement in DPR, and scores below 0 indicated the worsening of DPR.

#### DPR Measured by DDPRQ-10

DPR from the doctor's perspective was assessed using Difficult Doctor-Patient Relationship Questionnaire (DDPRQ-10), which has been used for the assessment of DPR in emergency care (28), primary care (29), and many other previous studies in China (30, 31). It is a doctor-rated scale to assess the degree of difficulty in their communications with patients. The scale consists of 10 questions rated on a 6-point Likert scale ranging from 1 (not at all) to 6 (a great deal), with a total score ranging from 10 to 60 and higher scores indicating poorer DPR.

#### Doctor's Trust in Patients

We used the Physician Trust Scale (TPS) compiled and revised by Liu in 2016 (32) (with a Cronbach's alpha of 0.93 in the validation sample), which was derived from the Physician Trust in the Patient Scale (PTPS) developed by Thom et al. (33) to measure the degree of doctors' trust in patients. It consists of 10 questions that are rated on a 5-point Likert scale ranging from 1 (strongly disagree) to 5 (strongly agree). The total score is the sum of the scores of the 10 questions, which ranges from 10 to 50. Higher scores indicate greater trust of doctors in patients.

#### Perceived Respect and Trust Before and During COVID-19

Respondents' perceived respect and trust from patients (perceived patients' respect/trust) before and during the COVID-19 pandemic was assessed through questions such as “How much respect/trust does the patient have for your profession before/ during the pandemic?” Respondents' perceived respect and trust from most people other than their patients (perceived most people's respect/trust) were measured through questions such as “How much respect/trust does most other people have for your profession before/ during the pandemic?” Each respondent was supposed to answer a total of eight questions, which were rated on a 5-point Likert scale ranging from 1 (very disrespectful/distrustful) to 5 (very respectful/trustful).

#### Workplace Violence Before and During COVID-19

Violence experienced by the respondents were assessed through four questions rated on a binary (yes-no) scale. The questions were “Have you experienced verbal/physical violence before/during the pandemic?” and “Have your colleagues experienced verbal/physical violence before/during the pandemic?” From the questions, eight variants could be obtained. The changes in workplace violence were calculated by taking the difference before and during the COVID-19 pandemic, and then classified into four categories: *higher level of violence*, s*ame level of violence, no violence*, and *lower level of violence*.

#### Important Elements That Will Impact DPR During the Pandemic

For this part, respondents should rate 10 items regarding the importance of factors that might impact the DPR during the pandemic, for example, “positive media reports on medical workers, such as the praise for their volunteering in Wuhan or the frontline of other areas” and “the national policy to provide free treatment for patients diagnosed with and suspected of COVID-19 infection.” All the items were rated on a 4-point Likert Scale ranging from 1 (negative influence) to 4 (*positive influence*).

#### Factors That Could Affect and Improve DPR in General

For this part, respondents were supposed to select five from twelve or nine items that might affect or improve DPR. The pool of items included “medical knowledge,” “communication,” “medical insurance,” “medical technology,” and “hospital management.” These items did not specifically target the period around the COVID-19 pandemic; the respondents should give their answers on the basis of their experience in recent years. See **Table 3** for details.

### Data Quality Control

To ensure the quality of the data, we performed quality control to further exclude unreliable responses. In this process, we needed to make sure questionnaires with logic verification error were eliminated, respondents only completed their questionnaires once regardless of what device they used (e.g., mobile phone, computer), and the minimum response time must be more than 3 min. Finally, respondents must enter a verification code upon the submission of their final responses.

### Statistical Analysis

One-way analysis of variance (ANOVA) was used to analyze the impact of ten respondent demographic variables with regard to DPR in recent years, DPR during the pandemic, and trust (see Table 1 for details), with each demographic variable as an independent variable and DPR and trust as dependent variables. The Bonferroni method was used for the correction of multiple comparisons in the ANOVAs, with the significance threshold set at *p* < 0.005. *Post-hoc* analyses for demographics were conducted using Tukey's test to find the variables that showed significant differences in the scores of DPR and trust. One-way ANOVA was also used to examine whether changes in workplace violence had an impact on DPR. The changes in workplace violence were used as an inter-subject variable, and the change in DPR was used as a dependent variable. Cochran's Q test was used to find factors that affected and improved DPR separately.

**Table 1:** Demographic characteristics and variables of doctor-patient relationship.

**Variables**		***N*(%) = 979**	**DPR**				**TPS**	
			**Recent years (SD)**	***p*-value**	**COVID-19 (SD)**	***p*-value**	**Mean (SD)**	***p*-value**
Gender	Female	639 (65.30%)	2.44 (0.91)	N.S.	3.60 (0.87)	N.S.	30.30 (4.79)	N.S.
	Male	340 (34.70%)	2.36 (0.97)		3.63 (0.93)		29.99 (5.08)	
Age		36.75 (8.14)						
Education	High school	26 (2.70%)	3.04 (1.15)	<0.01	3.96 (1.04)	N.S.	30.18 (4.73)	<0.01
	College	750 (76.60%)	2.40 (0.92)		3.63 (0.88)		34.15 (7.59)	
	Master's and above	203 (20.70%)	2.37 (0.89)		3.49 (0.89)		29.74 (4.88)	
Monthly Income	<50 k	149 (15.20%)	2.55 (1.10)	N.S.	3.65 (0.98)	N.S.	31.15 (6.43)	N.S.
	50–100 k	466 (47.60%)	2.40 (0.93)		3.59 (0.89)		30.32 (4.31)	
	100–200 k	286 (29.20%)	2.39 (0.83)		3.59 (0.86)		30.13 (4.33)	
	>200 k	78 (8.00%)	2.31 (0.93)		3.72 (0.79)		29.91 (4.72)	
Residency	City	891 (91.00%)	2.40 (0.92)	N.S.	3.61 (0.89)	N.S.	30.11 (4.80)	N.S.
	Town	51 (5.20%)	2.61 (1.10)		3.65 (0.96)		31.92 (6.92)	
	Village	37 (3.80%)	2.51 (0.90)		3.59 (0.86)		29.84 (3.24)	
Hospital level	Individual clinics	13 (1.30%)	2.38 (1.04)	N.S.	3.77 (0.83)	N.S.	31.14 (5.68)	<0.01
	Private	89 (9.10%)	2.28 (0.89)		3.70 (0.82)		34.00 (3.87)	
	Township	60 (6.10%)	2.43 (0.95)		3.67 (0.86)		29.92 (4.60)	
	County	175 (17.90%)	2.55 (1.02)		3.59 (0.96)		30.42 (5.88)	
	Prefecture	318 (32.50%)	2.43 (0.92)		3.60 (0.89)		29.72 (4.38)	
	Provincial and ministerial	324 (33.10%)	2.36 (0.89)		3.58 (0.88)		30.28 (4.57)	
Occupation	Clinician	565 (57.70%)	2.38 (0.90)	N.S.	3.55 (0.92)	N.S.	30.06 (4.87)	N.S.
	Logistic	10 (1.00%)	3.30 (1.42)		4.10 (0.99)		28.20 (5.35)	
	Management	58 (5.90%)	2.45 (1.03)		3.76 (0.71)		29.90 (3.85)	
	Medical Technician	109 (11.10%)	2.39 (0.89)		3.72 (0.84)		30.52 (5.25)	
	Nurse	237 (24.20%)	2.47 (0.96)		3.65 (0.85)		30.51 (4.99)	
Level	Entry	312 (31.90%)	2.48 (0.99)	N.S.	3.62 (0.91)	N.S.	30.46 (5.97)	N.S.
	Intermediate	413 (42.20%)	2.39 (0.92)		3.61 (0.90)		30.27 (4.34)	
	Senior	254 (25.90%)	2.38 (0.88)		3.59 (0.85)		29.73 (4.23)	
Experience	5 years and below	170 (17.40%)	2.41 (0.92)	N.S.	3.42 (0.96)	N.S.	29.55 (4.39)	N.S.
	6–10 years	242 (24.70%)	2.46 (0.96)		3.66 (0.92)		30.23 (4.20)	
	11–15 years	234 (23.90%)	2.32 (0.90)		3.57 (0.88)		31.18 (4.37)	
	16–30 years	276 (28.20%)	2.45 (0.93)		3.68 (0.82)		30.29 (6.47)	
	31 years and above	57 (5.80%)	2.46 (0.93)		3.77 (0.87)		30.48 (4.90)	
Pre-COVID Department	Department of Infectious Diseases	9 (0.90%)	2.78 (0.97)	N.S.	4.00 (1.00)	N.S.	31.78 (3.96)	N.S.
	Department of Psychiatry/Psychology	333 (34.00%)	2.38 (0.93)		3.61 (0.89)		30.13 (4.55)	
	Department of Traditional Chinese Medicine	11 (1.10%)	2.82 (1.08)		4.00 (0.63)		30.55 (4.01)	
	Emergency Department	14 (1.40%)	2.43 (1.09)		3.86 (1.17)		29.29 (3.85)	
	Gynecology and Obstetrics	39 (4.00%)	2.10 (0.55)		3.44 (0.88)		29.36 (5.24)	
	Internal Medicine	163 (16.60%)	2.36 (0.92)		3.54 (0.86)		30.71 (5.43)	
	Medical technical departments	74 (7.60%)	2.41 (0.95)		3.39 (0.98)		30.45 (4.61)	
	Others	140 (14.30%)	2.56 (0.97)		3.61 (0.86)		30.01 (4.75)	
	Pediatrics	42 (4.30%)	2.43 (0.91)		3.50 (0.97)		29.02 (4.05)	
	Pharmacy department	25 (2.60%)	2.52 (1.00)		3.76 (0.88)		31.92 (6.54)	
	Surgery	129 (13.20%)	2.44 (0.93)		3.80 (0.83)		30.02 (5.31)	
COVID Department	Fever Clinic	51 (5.20%)	2.57 (1.12)	N.S.	3.67 (0.97)	N.S.	29.65 (5.11)	N.S.
	Hubei Front-line Support	17 (1.70%)	2.29 (0.77)		3.65 (1.00)		29.71 (3.84)	
	Isolation and Observation Ward	45 (4.60%)	2.29 (0.79)		3.60 (0.84)		29.67 (3.42)	
	Nuclear Department	758 (77.40%)	2.43 (0.91)		3.59 (0.89)		30.21 (4.70)	
	Others	98 (10.00%)	2.33 (1.01)		3.73 (0.79)		30.88 (6.81)	
	Treatment Clinic	10 (1.00%)	2.40 (0.97)		3.70 (1.34)		28.20 (2.15)	

*All p-values are adjusted for Bonferroni correction*.

Three stepwise backward linear regression was used to analyze the important elements that affected DPR during the pandemic and might affect short-term and long-term DPR after the pandemic. Changes in respect, trust, verbal and physical violence, and other elements of DPR were used to predict DPR changes. In addition, change in DPR during the pandemic and the expected short-term change in DPR after the pandemic were also used as predictors for short-term and long-term DPR. All statistical significance levels were set at *p* < 0.05 (two-tailed) unless stated otherwise. All the data were analyzed with the use of R 4.0.3.

## Results

### Demographic Information

A total of 1,064 respondents completed the survey; 42 were excluded because of uncompleted questionnaire, another 32 were excluded because of responses that could not be logically verified by the platform, and 11 were excluded as their time of completion was shorter than the minimum time required, i.e., 3 min. The final sample consisted of 979 respondents, with a response rate of 92%. For the respondents included, the mean age was 36.75 years (SD = 8.14, range = 18–68 years), 65.30% were female (*N* = 639), 953 had a bachelor's degree or above (97.30%), and 891 lived in the urban area (91.00%). With regard to the level of hospital, respondents working in provincial hospitals accounted for the highest proportion (*N* = 324, 33.10%). Most of the respondents are clinicians (*N* = 565, 57.70%), and a large proportion of them held intermediate titles (*N* = 413, 42.20%). Other demographic characteristics are presented in Table 1.

### DPR Before and During the COVID-19 Pandemic

In this study, we found that the Cronbach's alpha of DDPRQ-10 was 0.43 for data before the COVID-19 pandemic and 0.18 for data during the COVID-19 pandemic, indicating poor reliability of the DDPRQ-10 in this sample. Therefore, we used general DPR questions (recent-year DPR, DPR around the COVID-19 period) for the analyses instead.

**Figure 1:**
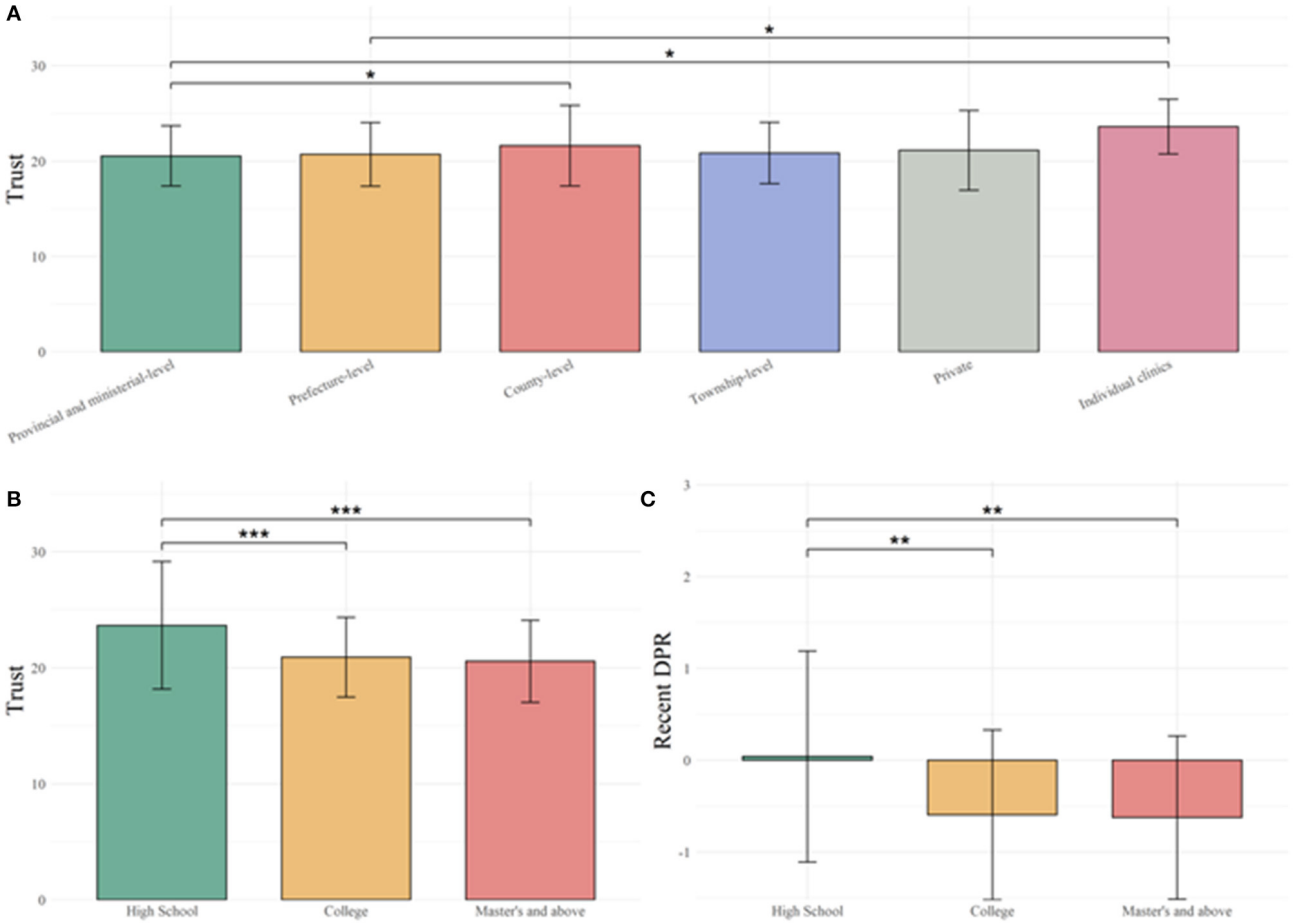
Change in perception of trust in medical services and doctor-patient relationship (DPR) before and during COVID-19. **(A)** Medical professionals working at prefecture-level and provincial and ministerial-level hospitals have lower trust in patients than those working in individual clinics. Furthermore, medical professionals working at provincial and ministerial-level hospitals have lower trust in patients than those working at county-level hospitals. **(B)** Medical professionals who have completed high school had higher trust in patients compared to those who have college or master's degree and above. **(C)** Medical professionals who have completed high school had positive change in DPR compared to medical professionals who have completed college or master's degree and above. * < 0.05, ** < 0.01, *** < 0.001.

One-way ANOVA revealed a significant effect of education on recent-year DPR [*F*_(2, 976)_ = 6.17, *p* < 0.001, η^2^ = 0.012] after Bonferroni correction. No significant difference was found in other demographic variables for recent-year DPR. Tukey's *post-hoc* test revealed that compared to high school education (M = 0.039, SD = 1.15), respondents with bachelor's degree (M = −0.60, SD = 0.93, *p* = 0.0018), master's degree and above (M = −0.63, SD = 0.89, *p* = 0.0017) had a lower score in recent-year DPR (Figure 1).

### Medical Professionals' Trust in Patients During the COVID-19 Pandemic

The Cronbach's alpha of TPS was 0.74 for the total score, indicating that it was adequate for analysis. One-way ANOVA revealed a significant effect of education [*F*_(2, 976)_ = 9.54, *p* < 0.001 η^2^ = 0.019] and the level of hospital [*F*_(5, 973)_ = 3.79, *p* = 0.0021, η^2^ = 0.019] on trust in patients after Bonferroni correction. No significant difference was found in other demographic variables for trust. Tukey's *post-hoc* test for the level of hospital revealed that compared to provincial hospitals (M = 29.70, SD = 4.38), respondents who worked at county-level hospitals (M = 31.10, SD = 5.68, *p* = 0.022) and individual clinics (M = 34.00, SD = 3.87, *p* = 0.023) had higher levels of trust in their patients. And compared to respondents working at prefecture-level hospitals (M = 29.90, SD = 4.60), those working at individual clinics (*p* = 0.037) had higher levels of trust in their patients. Tukey's *post-hoc* test for education levels also showed that compared to high school education (M = 34.20, SD = 7.59), respondents with bachelor's degree (M = 30.20, SD = 4.73, *p* < 0.001), and master's degree and above (M = 29.7, SD = 4.88, *p* < 0.001) had lower trust in their patients (Figure 1).

### Perceived Respect and Trust Before and During COVID-19

Mixed-factorial ANOVA revealed a main effect of perceived self-/other-trust [*F*_(1, 3911)_ = 54.27, *p* < 0.001, η^2^ = 0.014] and timepoint [*F*_(1, 3911)_ = 41.45, *p* < 0.001, η^2^ = 0.011]. However, no significant trust × timepoint interaction was found [*F*_(1, 3911)_ = 0.55, *p* > 0.05]. It was also found that perceived other-trust (M = 3.66, SD = 0.64) was higher than perceived self-trust (M = 3.59, SD = 0.63). Perceived trust was greater during the COVID-19 pandemic (M = 3.75, SD = 0.64), as compared to that before the pandemic (M = 3.50, SD = 0.60).

Mixed-factorial ANOVA revealed a main effect of perceived self-/other-respect [*F*_(1, 3911)_ = 225.56, *p* < 0.001, η^2^ = 0.058] and timepoint [*F*_(1, 3911)_ = 88.71, *p* < 0.001, η^2^ = 0.023]. However, no significant perceived respect × timepoint interaction was found [*F*_(1, 3911)_ = 2.87, *p* > 0.05]. Perceived other-respect (M = 3.50, SD = 0.64) was higher than perceived self-respect (M = 3.44, SD = 0.71); and perceived respect was greater during the COVID-19 pandemic (M = 3.69, SD = 0.67), as compared to that before the pandemic (M = 3.25, SD = 0.62).

### Violence Against Doctors Before and During COVID-19

Respondents reported that verbal violence against them and their colleagues decreased by 56.68 and 82.80%, respectively, during the pandemic, and physical violence against them and their colleagues also decreased by 85.96 and 76.11%, respectively. One-way ANOVA revealed no significant effect of physical or verbal offenses toward the respondents or their colleagues on the DPR (*P*s > 0.05).

### Factors That Affect and Improve DPR

Cochran's Q test revealed the significant factors that affected DPR [Q_(11)_ = 3,997.83, *p* < 0.001]. The top five factors were high expectations for doctors and the opinion that doctors should know everything (87%), various causes of low mutual trust between doctors and patients (76%), patient's lack of knowledge (69%), negative reports or misinformation of medical and pharmaceutical industries by the media (66%), and difficulty in visiting a doctor and high cost for the consultation (58%).

Cochran's Q test revealed the significant factors that improved DPR [Q_(8)_ = 3,304.53, *p* < 0.001]. The top five factors were the improvement of medical legislations (84%), good doctor-patient communication (84%), basic medical knowledge for patients (82%), media responsibility (75%), and medical insurance (57%). See Table 2 for details.

**Table 2:** Factors that affect and improve doctor-patient relationship.

**Factors**	**Description**	**Percentage**
Factors that affect DPR	Various reasons that lead to low trust between doctors and patients	76%
	It is difficult and expensive to see a doctor	72%
	The lack of knowledge about diseases, treatment process, and prognosis for the public	58%
	Patients' high expectations of doctors, thinking that doctors know everything	87%
	Public impression of doctors becomes less favorable because of occasional bribery and rebates	17%
	Negative media reports or misinformation of medical and pharmaceutical industries	66%
	Difficulty in addressing medical disputes through formal channels, and lack of penalty for violence against medical workers	44%
	Limitation in medical technology and service quality	13%
	Issues in doctor-patient communication (e.g., inadequate communication due to very tight schedules of doctors, etc.)	27%
	Poor hospital management, and improper handling of medical disputes	8%
	Low medical insurance reimbursement ratio	14%
	Others	2%
Factors that improve DPR	Extensive public education of healthcare knowledge as well as the limitation of modern medicine	82%
	Improvement in medical technology and service quality	53%
	Improvement in communication between doctors and patients, such as reducing the work load of doctors and nurses to allow more time for communication	84%
	Improvement in media objectivity to reduce misinformation	75%
	Introduction of legal approaches to solve medical disputes	84%
	Improvement in the hospital security system and increase in the coverage of medical insurance	57%
	Improvement in hospital management, strengthening of medical ethics	21%
	Establishment of a good public impression of medical staff	5%
	Others	2%

### Factors Predicting Changes in DPR After the Pandemic

Backward stepwise linear regression for changes in DPR during the pandemic revealed a significant model at *F*_(9, 969)_ = 21.17, *p* < 0.001, and *R*^2^ = 0.16. Better understanding of the work of medical professionals (B = 0.33, *p* < 0.001), inconvenience in medical consultation during the pandemic (B = 0.070, *p* < 0.001), and medical professionals' trust in patients (B = 0.011, *p* = 0.023) were found positively associated with the DPR during the pandemic.

Backward stepwise linear regression revealed a significant model for short-term DPR after the pandemic [*F*_(7, 971)_ = 54.98, *p* < 0.001, *R*^2^ = 0.28]. Better understanding of the work of medical professionals (B = 0.20, *p* < 0.001), inconvenience in medical consultation during the pandemic (B = 0.054, *p* = 0.038), measures to encourage and care for medical professionals (B = 0.14, *p* < 0.001), public nervousness and panic during the pandemic (B = 0.048, *p* =0.0012), and change in DPR during the pandemic (B = 0.26, *p* < 0.001) were associated with higher expectation of short-term DPR in the future.

Backward stepwise linear regression revealed a significant model for expected long-term DPR after the pandemic [*F*_(10, 968)_ = 37.83, *p* < 0.001, *R*^2^ = 0.29]. Positive media reports on medical staff (B = 0.17, *p* < 0.001), inconvenience in medical consultation during the pandemic (B = 0.043, *p* = 0.018), disproportionate frontline and insufficient hospital staff (B = 0.048, *p* = 0.011), medical professionals' trust in patients (B = 0.014, *p* < 0.001), change in perception of physical harm to doctors (self; B = 0.087, *p* = 0.012), and expected short-term DPR after the pandemic (B = 0.42, *p* < 0.001) were associated with higher expectation of long-term DPR in the future. Details are shown in Table 3.

**Table 3:** Regression weights of short-term and long-term doctor-patient relationship.

**Variables**	**DPR change during the pandemic (B)**	**Expected short-term change (B)**	**Expected long-term change (B)**
**Importance of elements of DPR**			
Better public understanding of the work of medical professionals	0.33^***^	0.20^***^	−0.061
Awareness of limitations of modern medicine	0.060	0.054^*^	–
Positive media reports on medical staff	0.092	–	0.17^***^
Measures to encourage, and care for, medical professionals	–	0.14^***^	–
Inconvenient process of medical consultation during the pandemic	0.070^***^	–	0.043^*^
Disproportionate frontline staff and insufficient hospital staff	–	–	0.048^**^
Public nervousness and panic during the pandemic	0.038	0.048^**^	0.031
Free online consultations, psychological hotlines, and other activities	−0.077	–	–
**Perception of respect and harm**			
Change in self-perceived respect for doctors	0.047	–	–
Change in other-perceived respect for doctors	–	0.035	–
Change in perceived verbal harm to doctors (self)	–	–	0.032
Change in perceived physical harm to doctors (self)	0.069	–	0.087^*^
**DPR changes**			
Changes in DPR during the pandemic	–	0.26^***^	0.052
Expected short-term change in DPR	–	–	0.42^***^
**Trust**			
TPS	0.011^*^	0.0068	0.014^***^
Model *R^2^*	0.16^***^	0.28^***^	0.29^***^

* *p < 0.05*,

** *p < 0.01*,

*** *p < 0.001*.

## Discussion

To our knowledge, this is the first quantitative empirical study on the perception of DPR among Chinese medical workers during the pandemic. This study revealed that Chinese medical workers were optimistic about the DPR during the COVID-19 outbreak. We also examined how their perceptions of DPR were impacted by multiple factors, such as demographic characteristics and changes in healthcare systems in response to the pandemic. In addition, several significant predictors for DPR after the pandemic were also found.

Consistent with the mainstream media report of doctor-patient interaction, the participants in the present study experienced a better DPR during the outbreak, reporting more respect and trust and less violence from the public, which supported our hypothesis that Chinese medical professionals might report improved DPR during the COVID-19 pandemic. In their fight against the virus, both medical workers and patients were supportive and understanding to each other (34). Meanwhile, medical workers have received national recognition and gained public support and respect during the outbreak, which further improved their image and social status (23, 35). Furthermore, policies were developed to provide incentives to medical workers and protect them in all aspects, including psychological health services, daily needs, work-related injury compensation, subsidies and allowances, etc. (36, 37). All of the above factors contributed to a better DPR perceived by Chinese medical workers.

Another finding was the differences in the influence of levels of education and hospitals on trust and DPR. Medical workers with higher level of education (bachelor's and master's degree) had lower trust and DPR than those who received only high school education. Similar results were found in other studies, where education is significantly and negatively correlated with trust and DPR (31, 38). The present study also found that medical workers from a higher level of hospital had lower trust in patients than those from a lower level hospital, which was consistent with another study that reported a substantial influence of the level of hospital on DPR from the doctor's perspective (39). A possible reason for this counterintuitive finding is that most highly educated doctors work in higher-level hospitals, which are usually associated with higher workloads (40), greater pressure, as well as more medical disputes (41). The potential stressors may result in decreased enthusiasm of medical staff and negative views toward DPR and trust (42).

Our study also identified some predictors of DPR over time from medical workers' perspective, including patients' understanding of medical professionals, patients' awareness of the limitation of modern medicine, patients being supportive to medical professionals, positive media report about medical staff, medical professionals' trust in patients, the reduction of physical and verbal violence against doctors, etc. Notably, patients' understanding of medical professionals was significantly positively correlated with perceived DPR during and shortly after the pandemic, which was in line with previous reports, which demonstrated the needs of medical professionals for public understanding of the challenges they faced (43). In the fight against the pandemic, medical workers in China faced tremendous stress, burnout, physical health risks, psychological health issues, etc. (44, 45). Through the outbreak, people began to realize the limitation of modern medicine, and began to empathize and support medical workers, which in turn encouraged the medical professionals in their works and improved their perception of DPR. Patients' basic health knowledge is also important (46), as it helps them understand the limitation of medicine and improve their communication with doctors (47). Factors predicting long-term DPR after the pandemic are also critical, as they influences the public opinion toward medical workers in the long run. For example, positive media report about medical staff and positive press coverage about doctors could potentially improve public trust in doctors (24, 47). In addition, our study found that previous perception of DPR is positively correlated with expected short-term DPR, which was found to be positively associated with long-term DPR. From this result, we can be optimistic about the DPR in the future despite the challenges medical workers are faced with. As DPR is changing gradually over time, the findings in our study might be hints for the development of public health policies on the basis of status quo.

### Implications for Public Health

The results of this study have great implications for long-term development of policies and medical systems for future medical emergencies. First, our findings suggested that the image of medical professionals has been improved through the pandemic. Previously, due to various factors such as information asymmetry, some people in China might have an unfavorable impression of medical professionals in recent years (48). However, during the pandemic, the heroic deeds of medical professionals are seen and recognized by the public. Second, we also found that the management of healthcare resources and contingency plans are important for medical emergencies (49, 50). Strategic leadership, adaptiveness, communication, information transparency, responsibility, and the professionalism will significantly benefit everyone in the country during a public health emergency (49, 51, 52). The interconnected system through digital means, as well as technology used to aid management and treatment will boost the capability of medical system to provide interventions. This can also improve the trust in the medical system, leading to better DPR. Finally, the government should seek to improve the healthcare education for the public to narrow the knowledge gap between medical workers and the public. With better understanding of doctors' work, patients might be more compliant with their treatment (46), show more respect to and trust in their doctors (24, 47), which might lead to better DPR in the long run. Overall, the current COVID-19 crisis has affected DPR in a variety of aspects, and we need to utilize the opportunities brought about by the current health issue to improve the DPR.

### Limitations

Despite the insights of our study, there are a few limitations. First, this is a retrospective study on medical professionals' perception of DPR before and during the pandemic. Therefore, it is difficult to make sure whether these factors will still be predictors in the future. A longitudinal study might be needed to identify predictors for long-term changes in DPR. Second, the reliability of the DDPRQ-10 was found to be poor in this study; thus, we needed to use general questions regarding DPR. Future works are needed to develop valid and reliable questionnaires for the assessment of the relationship between two parties. Finally, regarding expected short-term and long-term changes in DPR, we did not provide a definition of the two terms; therefore, the respondents may answer relevant questions based on their own perception. Despite these limitations, our study had enough power and sample size to identify important factors and predictors of DPR, which is of great importance in the public health and policy making in China.

## Conclusions

DPR is important for patient outcomes, especially during a public health emergency such as the COVID-19 pandemic. Understanding DPR from the doctor's perspective is crucial for medical administration, hospital management, and patient care. Our study showed that Chinese medical workers were optimistic about DPR during the COVID-19 outbreak. Demographic characteristics such as education and the level of hospital they are working at were associated with DPR and trust. We also identified predictors for changes in DPR during the pandemic and in short term and long term after the pandemic. These factors have broad implications for policy making and medical resource management, and may help improve the medical system and doctor-patient relationship in the future.

## Data Availability Statement

The raw data supporting the conclusions of this article will be made available by the authors, without undue reservation.

## Ethics Statement

The studies involving human participants were reviewed and approved by the Ethics Committee of The Second Xiangya Hospital of Central South University. The patients/participants provided their written informed consent to participate in this study.

## Author Contributions

The study was conceptualized by YZ and TL. The data was collected and organized by YZ. Data were analyzed and interpreted by YZ and WY. The manuscript was drafted by YZ and revised by TL, XW, DY, YM, and QW. All authors have read and approved the final manuscript.

## Funding

This study was supported by the National Key R&D Program of China (2017YFC1310400), the Technology innovation guidance plan of Hunan province (2017SK50315), the Project of Hunan Health and Family Planning Commission (B20180484), and the Science and Technology Plan of Changsha City, Hunan Province (kq2004106).

## Conflict of Interest

The authors declare that the research was conducted in the absence of any commercial or financial relationships that could be construed as a potential conflict of interest.

## Publisher's Note

All claims expressed in this article are solely those of the authors and do not necessarily represent those of their affiliated organizations, or those of the publisher, the editors and the reviewers. Any product that may be evaluated in this article, or claim that may be made by its manufacturer, is not guaranteed or endorsed by the publisher.
